# Autologous thyroid cartilage graft implantation in medialization laryngoplasty: a modified approach for treating unilateral vocal fold paralysis

**DOI:** 10.1038/s41598-017-05024-6

**Published:** 2017-07-06

**Authors:** Ming-Shao Tsai, Ming-Yu Yang, Geng-He Chang, Yao-Te Tsai, Meng-Hung Lin, Cheng-Ming Hsu

**Affiliations:** 1 Department of Otolaryngology – Head and Neck Surgery, Chiayi Chang Gung Memorial Hospital, Chiayi, Taiwan; 2 Center of Excellence for Chang Gung Research Datalink, Chiayi Chang Gung Memorial Hospital, Chiayi, Taiwan; 3 Graduate Institute of Clinical Medical Sciences, College of Medicine, Chang Gung University, Taoyuan, Taiwan; 4 School of Traditional Chinese Medicine, College of Medicine, Chang Gung University, Taoyuan, Taiwan

## Abstract

Medialization laryngoplasty is the standard surgical treatment for unilateral vocal fold paralysis. This study presents a modified approach in which a thyroid cartilage graft is implanted in medialization laryngoplasty. 22 patients who underwent this approach were included in the study. The results revealed that glottal incompetence and vocal performance were markedly improved following surgery, and the follow-up period ranged from 6 to 74 months (mean, 21.4 months). Acoustic analysis revealed significant improvements in the maximum phonation time (from 3.51 to 7.89 seconds, p < 0.001), F0 (from 221.7 to 171.0 Hertz, p = 0.025), and jitter (from 7.68 to 3.19, p < 0.001). Perceptual assessment revealed a significant decrease in voice grading (from 2.59 to 1.41, p < 0.001), roughness (from 1.82 to 1.23, p = 0.004), and voice breathiness (from 2.55 to 1.23, p < 0.001). None of the patients exhibited severe wound infection, tissue rejection, or other complications attributed to the surgical procedure. In conclusion, autologous thyroid cartilage implantation in medialization laryngoplasty medializes the vocal cord, minimizes the glottal gap, and improves the voice of patients with vocal fold paralysis. This procedure is characterized by simplicity, safety, and acceptable results.

## Introduction

Unilateral vocal fold paralysis (UVFP) occurs when one vocal fold is paralyzed in the paramedian or lateral position with very limited movement^1^. UVFP is usually secondary to neurological injury. It may be caused by several etiologies, which can be divided into three categories: surgical (e.g., thyroid, chest, and brain surgeries), nonsurgical (e.g., tracheal intubation, lung cancer, and cerebrovascular disease), and idiopathic^2–6^. Glottal incompetence caused by UVFP prevents the vocal folds from closing completely and impairs phonation and swallowing^7^. Without adequate compensation, patients with UVFP present with dysphonia and dysphagia that includes aspiration. Frequent aspiration can lead to pneumonia or other pulmonary complications with potentially life-threatening consequences^8, 9^.

The current treatment for UVFP includes medialization laryngoplasty with or without arytenoid adduction (AA)^10, 11^, injection laryngoplasty^12^, laryngeal reinnervation^13^, adduction arytenopexy, and cricothyroid subluxation^14^. A 6–12-month observation period is generally suggested before permanent medialization laryngoplasty^15^. During the 6–12-month waiting period, vocal fold injection with autologous fat or hyaluronic acid is a viable treatment option with favorable short-term outcomes; however, this treatment option is less suitable for patients with large posterior glottal gaps, and its long-term benefit is controversial^16–21^. Moreover, laryngeal reinnervation has been reported to be less favorable outcomes in older patients and those with atrophy of the laryngeal muscle and a longer interval between injury and reinnervation^22, 23^. Therefore, medialization laryngoplasty is a standard treatment when long-lasting improvement is required^21, 24, 25^. Isshiki type I thyroplasty is the standard medialization laryngoplasty technique, which has been used since the 1980s and involves silastic implantation through a thyroid cartilage window to augment the vocal fold^26–28^. Isshiki type I thyroplasty with nonresorbable biomaterials, such as silicone, Gore-Tex, hydroxyapatite, titanium, and expanded polytetrafluoroethylene, has also been performed in the past few decades^29–34^. Although these materials are effective for vocal improvement, they are associated with disadvantages such as graft rejection and some potential complications. Therefore, highly biocompatible autologous or homologous grafts, which are cost free, easily available, and associated with lower rejection rates compared with alloplastic implants, are required^35^.

Autologous grafts are freely available, have no risk of tissue rejection, and are highly suitable for medialization laryngoplasty. The cartilage is a preferred autologous graft material, because of its easy availability, low absorption rate, high biocompatibility, and minimal tissue reaction or fibrosis with minimal shrinkage capacity^36^. In 1955, Arnold treated vocal fold paralysis with autologous cartilage and bone dust injections^37^. Subsequently, in an animal study, vocal fold paralysis was treated with elastic auricular cartilage injection^38^. Recently, human autologous cartilage, such as nasal or auricular cartilage, has been used as potentially permanent implant materials for injection laryngoplasty^39, 40^. The successful outcomes of autologous cartilage graft implantation suggest that these grafts can be used in medialization laryngoplasty.

Although autologous cartilage grafts from the nasal septum or auricle are considered safe and effective for augmentation, this type of graft may be too fragile, and its implantation requires the creation of another external skin wound, which would cause more postoperative pain. Therefore, the thyroid cartilage seems more suitable for implants in medialization laryngoplasty, and only one surgical wound is created. The thyroid cartilage, the largest cartilage of the larynx, is composed of two fused plates of hyaline cartilage that protect the anterior wall of the larynx. In 1915, Payr used an anteriorly pedicled thyroid cartilage flap to medialize the vocal fold^41^. However, this method did not gain popularity because of some disadvantages regarding technique and its limited effect^41^. Opheim (1955), Parker (1955), Sawashima (1968), Smith (1972), Kamer and Som (1972), and Tucker (1979, 1983) continued to improve medialization laryngoplasty techniques using thyroid cartilage grafts^41, 42^. In this study, we present a modified approach that has not been previously described in other articles. We conducted a pilot study to demonstrate the surgical technique of implantation of the thyroid cartilage for glottal incompetence; however, the previous study included only six patients with UVFP^43^. Therefore, this study investigated functional outcomes after medialization thyroplasty using autologous thyroid cartilage implants for treating UVFP.

## Methods

### Patients

We reviewed the medical records of 25 consecutive patients with UVFP who were managed with the Isshiki type I thyroplasty technique using autologous thyroid cartilage implants with or without AA from January 2012 to March 2016. Three patients were excluded from the study because of incomplete outcome data. Finally, 22 patients were included. These patients were examined at 1 or 2 weeks preoperatively and were followed up at 1, 3, and 6 months postoperatively. Preoperative and postoperative vocal functions were analyzed. Laryngostroboscopy with acoustic analysis and perceptual assessment was performed. Information on age, sex, cause of UVFP, side of UVFP, position of the paralyzed true vocal fold, symptoms, preoperative severity of dysphonia, postoperative functional outcomes, and complications was collected. Informed consent was obtained from all patients after discussion of surgical outcomes and possible complications.

Patients with external laryngeal trauma, uncontrolled malignancy of the head or neck region, neoplastic disease involving the larynx, and severe laryngeal stenosis were excluded. Patients with central nervous systemic disease and those with inadequate cardiac or lung function who are unsuitable for this procedure were also excluded. This study was performed in the Department of Otolaryngology – Head and Neck Surgery, Chiayi Chang Gung Memorial Hospital, Chiayi, Taiwan. This study was approved by the Ethics Committees of Chang Gung Memorial Hospital (CGMH-IRB No. 100-2757B) and the methods in this clinical retrospective study were carried out in accordance with the approved guidelines and regulations. Informed consent was obtained from all subjects.

### Surgical procedures

**Figure 1:**
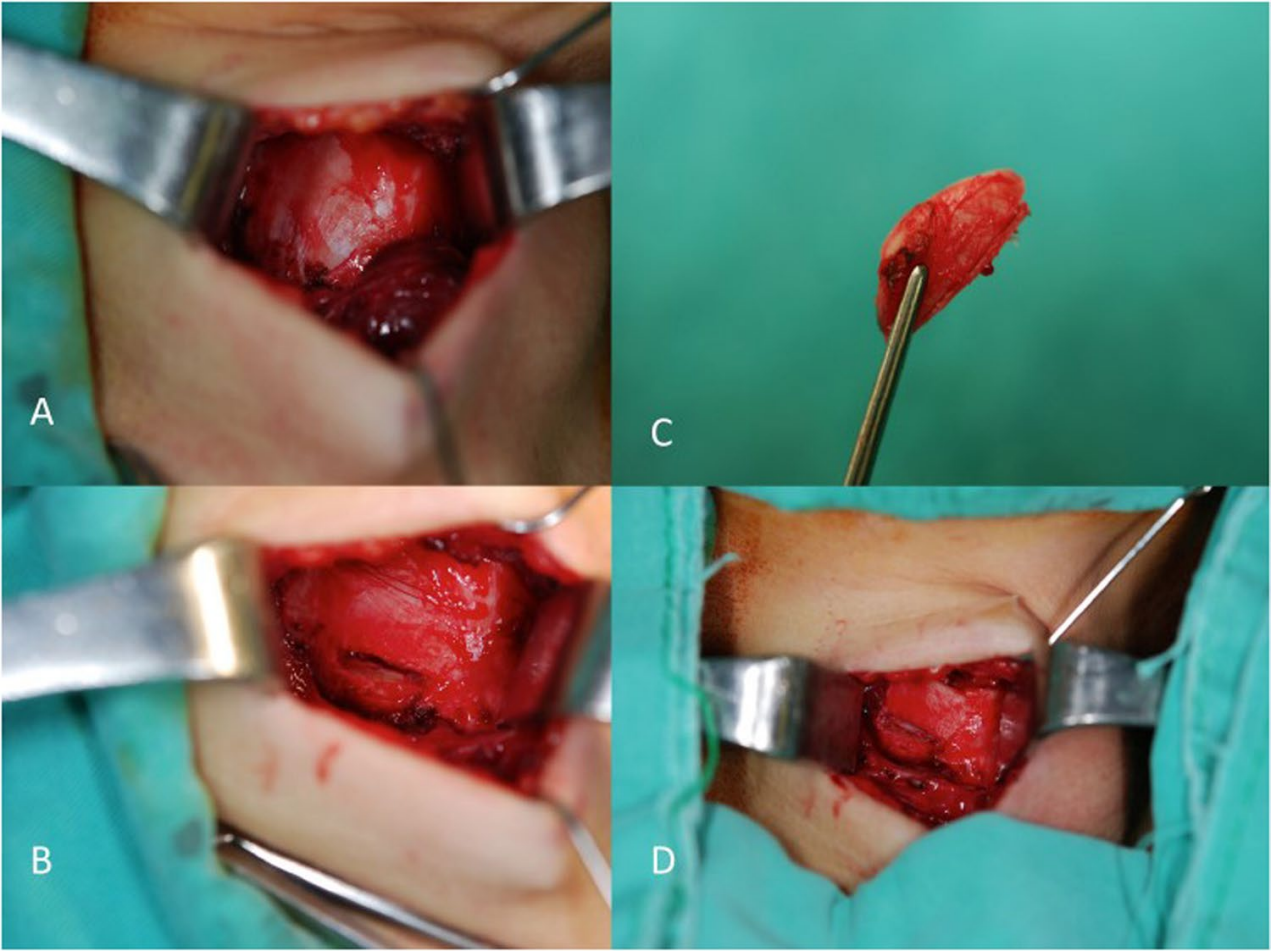
Illustration of surgical procedure. (**A**) Lesion side of the thyroid cartilage was exposed. (**B**) Creation of a narrow thyroplasty window. (**C**) A lunar-shaped cartilage graft. (**D**) The graft was inserted and then pressed into the window.

Medialization laryngoplasty using thyroid cartilage implants was performed under intravenous sedation and local anesthesia. With patients in the supine position, a 4- to 5-cm horizontal incision along the skin crease was made on the lesion side of the thyroid cartilage, and strap muscles were then dissected and pulled laterally to expose the ipsilateral thyroid cartilage from the upper to lower margins (Fig. 1A).

Marking the border of the thyroid window was performed before graft harvesting because once the graft was taken, the landmark was not easily identified. The midline of the thyroid cartilage spine was identified. A mark was placed at the third quarter of the midline of the spine through electrocautery. Another mark parallel to the initial mark was placed 5 mm laterally for women and 7 mm laterally for men, which defined the anterior borders of the thyroid cartilage window (usual width = 3–5 mm). The posterior window border was located immediately anterior to the oblique line; thus, the length of the thyroid cartilage window was approximately 15–18 mm. The inferior window border was located approximately 5 mm superior to the lower margin of the thyroid cartilage to prevent fracturing. Subsequently, the rectangular window was incised using a number 15 blade (Fig. 1B). For calcified cartilage, a small oscillating saw was used for superior and inferior borders, and a curved clamp was used to remove the remaining cartilage. A mucosal elevator was used to separate the underlying perichondrium from the cartilage, thus creating a space suitable for the cartilage implant.

Before taking the graft, we tested patients’ voice by pressing the soft tissue with the mucosal elevator through the window. While the patients phonated a long “e” sound, the surgeon pressed the vocal fold toward the midline. The depth pressed that achieved the best voice was the ideal height of the implant. This testing was termed “intraoperative testing,” helping the operator to determine the proper size of the graft. The graft was marked to confirm that the graft’s height was slightly (2–3 mm) longer than the ideal height of the implant and that the graft’s length was longer then the window’s length. The upper portion of the thyroid cartilage close to the laryngeal prominence was excised as a lunar-shaped cartilage graft (Fig. 1C). Hemostasis was carefully performed using bipolar cautery and suture ligation to avoid hematoma and tissue swelling. To smoothen the surface of the graft, the sharp edge was tailored using scissors.

**Figure 2:**
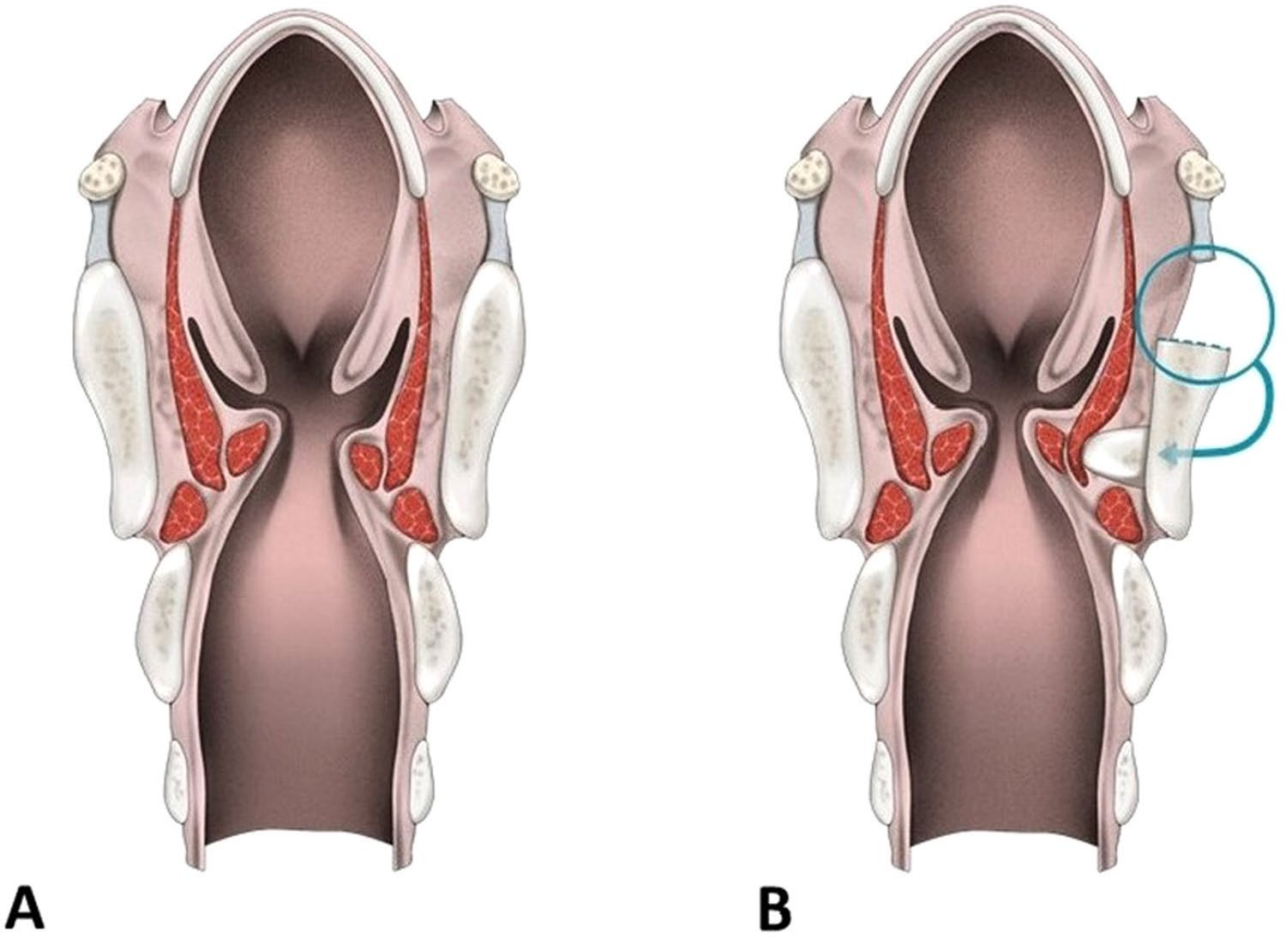
Schematic illustration of the coronal view of the larynx. (**A**) Right vocal fold was paralyzed with glottal insufficiency. (**B**) The graft was inserted into the window, and the paralyzed vocal fold was medialized. The authors would like to thank Biao-Wei Chen for drawing Fig. 2.

The cartilage was grasped using forceps, and the partial cartilage graft was then inserted obliquely into this window with the curved side facing the vocal fold. The remaining cartilage was held with forceps and pressed into the window using the mucosal elevator. The cartilage slid into the window and was locked tightly without suturing (Fig. 1D). Figure 2A and B illustrates the coronal view of the larynx and shows how the graft is inserted into the thyroid cartilage window to medialize the paralyzed vocal fold. Because the cartilage implants are vulnerable to dislocation, the window must be smaller than the thyroid cartilage, and its height must closely fit the cartilage thickness. A closely matched window increases cartilage stability.

Fine tune adjustment was performed by carefully downsizing the cartilage. For patients who still had a glottal gap and hoarseness of voice after graft implantation, simultaneous AA was performed to achieve optimal voice results. In most surgeries, we only needed to downsize the cartilage because cartilage slightly larger than its ideal size was used. At the end of the surgery, a folded Gelform was placed in the window to prevent dislocation and hematoma. After primary suturing, a closed wound drain consisting of perforated tubing was connected to a portable vacuum unit. The tubing exit site was treated as an additional surgical wound; the drain was typically sutured to the skin. The closed wound drain was usually removed within 3 days. In most cases, postoperative antibiotics were not required. A demonstration video titled “surgical procedure” has also been provided. The twelve key steps of surgical procedure are presented as supplementary information titled “Figure S1”.

### Voice analysis

Preoperative (1–2 weeks before surgery) and postoperative (3 months after surgery) vocal functions were recorded and analyzed. Laryngeal strobovideoscopy was performed using a Kay Elemetrics Stroboscopy Unit (Model, Lincoln Park, NJ, USA). The recorded data were evaluated in a blinded manner by a speech pathologist skilled in voice training and by an otolaryngologist. The acoustic parameters automatically recorded were the average fundamental frequency (F0) in Hertz, jitter, shimmer, and noise-to-harmonic ratio (NHR). Jitter is the cycle-to-cycle variation of the fundamental frequency. Shimmer is expressed as the variability of the peak-to-peak amplitude in decibels^44^. NHR is a measure for quantifying the amount of additive noise in the voice signal to evaluate a dysphonic voice^45^. These acoustic parameters were measured using a Computerized Speech Laboratory (core model CSL 4500, KayPentax, Lincoln Park, NJ, USA). The duration parameter recorded was the maximum phonation time (MPT). A speaker’s MPT was defined as the best attempt in seconds to maximally prolong the vowel /a/ at a comfortable intensity. The MPT was measured using a stopwatch. Pre- and postoperative perceptual assessments were completed according to the GRBAS scoring system (G = grade, R = roughness, B = breathiness, A = asthenia, and S = strain; 0 = normal, 1 = mild, 2 = moderate, and 3 = severe). The rating was made on a short standard passage. The mean of two values recorded by the speech pathologist and otolaryngologist was used for analysis. If a difference of more than 2 points was noted between the two GRBAS scores, a reevaluation was required.

### Voice satisfaction survey

All patients in this study were surveyed on their voice satisfaction (“satisfied” or “not satisfied”) six months after thyroid cartilage graft implantation.

### Statistics

Summary descriptive statistics are presented as the mean ± standard deviation for continuous variables. The Wilcoxon singed-rank test was used for nonparametric analysis of ranked data. Changes in the scores and data preoperatively and postoperatively were analyzed using SPSS software for Windows, Version 13.0 (SPSS, Chicago, IL, USA). All statistical analyses were performed at a two-sided 5% level of significance.

## Results

### Functional outcomes

**Figure 3:**
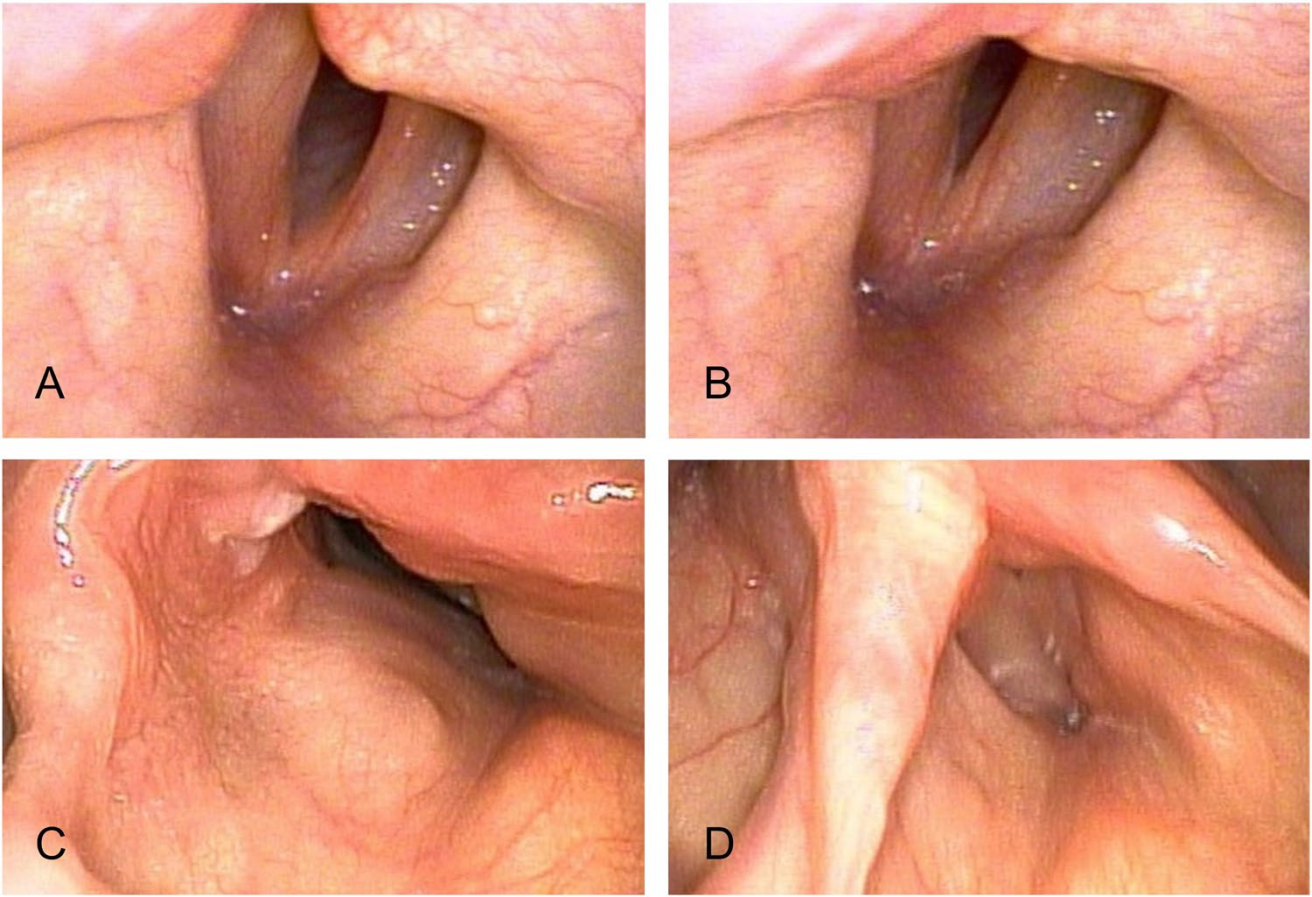
Endoscopic view of the larynx in a 40-year-old man with left vocal paralysis before (**A**, inhalation; (**B**) phonation) and after (**C**, inhalation; **D**, phonation) laryngoplasty with thyroid cartilage implantation. The glottal closure markedly improved.

The patients’ characteristics are presented in Table 1, and all underwent medialization laryngoplasty using autologous thyroid cartilage implants. The causes of UVFP were idiopathic in 3 and postsurgical trauma in 19 patients. The median onset time of vocal paralysis was 10 months. Before surgery, all patients had husky voices with a paralyzed vocal fold and phonatory gaps for more than 6 months. Figure 3 shows the preoperative glottal gap at rest (Fig. 3A) and that during phonation (Fig. 3B).

**Table 1:** Patient characteristics.

Variable	n	(%)
**Mean age (years, range)**	57.1	(33–84)
**Gender**		
Male	13	(59.1)
Female	9	(40.9)
**Cause of paralysis**		
Surgical	19	(86.4)
Non-surgical	0	(0)
Idiopathic	3	(13.6)
Median onset duration (months, range)	10	(6–24)
**Site of paralysis**		
Left	20	(90.9)
Right	2	(9.1)
**Type of surgery**		
TCI with AA	6	(27.2)
TCI without AA	16	(72.7)

*Abbreviations: TCI = Thyroid cartilage implantation; AA = Arytenoid adduction.

Postoperative follow-up time ranged from 6 to 74 months (mean, 21.4 months). Videostroboscopy, acoustic analysis, and perceptual assessment were conducted 3 months postoperatively. Figure 3C,D respectively shows the postoperative glottal gap at rest and that during phonation. Videostroboscopy revealed that, after surgery, all patients achieved markedly postoperative improvements in glottal closure, mucosal wave, and amplitude. No additional morbidity or complication was attributed to the surgical procedure.

### Objective voice parameters

The acoustic analysis results are summarized in Table 2. MPT and jitter significantly improved after surgery (Table 2). Moreover, postoperative F0 was significantly lower than preoperative F0. Postoperative shimmer and NHR decreased without statistical significance.

**Table 2:** Comparison of speech and voice parameters recorded preoperatively and postoperatively.

Parameters	Preoperative (mean ± SD)	Postoperative (mean ± SD)	*p*-value
**Acoustic analysis**			
F0 (Hertz)	221.7 ± 82.9	171.0 ± 59.1	0.025
Jitter	7.68 ± 5.56	3.19 ± 1.55	<0.001
Shimmer	1.11 ± 0.66	0.78 ± 0.47	0.059
MPT (second)	3.51 ± 1.60	7.89 ± 4.04	<0.001
NHR	0.39 ± 0.31	0.26 ± 0.30	0.156
**Perceptual assessment**			
Grade	2.59 ± 0.50	1.41 ± 0.85	<0.001
Roughness	1.82 ± 0.66	1.23 ± 0.61	0.004
Breathiness	2.55 ± 0.60	1.23 ± 0.92	<0.001
Asthenia	1.32 ± 0.72	0.86 ± 0.94	0.079
Strain	1.05 ± 1.09	0.64 ± 0.85	0.172

### Perceptual assessment

Preoperative and postoperative perceptual assessments were conducted according to GRBAS scoring, which revealed a significant decrease in voice grade, roughness, and breathiness (Table 2). Asthenia and strain decreased without statistical significance.

### Voice satisfaction

Of the 13 male patients, 11 patients felt satisfied (11/13, 84.6%), whereas 5 of the 9 female patients felt satisfied with the results (5/9, 55.6%).

## Discussion

Autologous thyroid cartilage graft implantation in medialization laryngoplasty is a modified approach for treating UVFP, and this approach achieves significant postoperative improvements both objectively in voice parameters and subjectively in perceptual assessments. Moreover, in this study, none of the 22 patients with UVFP exhibited a worsened condition in at least 6 months of follow-up, and none required a second medialization procedure at the mean follow-up time of 21.4 months. Dislocation did not occur because a narrow window was created to increase implant stability without excess mobility.

Our modified approach has some differences from previous graft harvesting and placement techniques. Payr made a U-shaped incision with a pedicled cartilage flap, and Smith used the upper portion of a contralateral alar cartilage graft. We used the upper portion of the ipsilateral thyroid cartilage close to the laryngeal prominence as a lunar-shaped cartilage graft, which created a smaller incision^41^. Parker inserted a graft into the space between the soft tissue and thyroid cartilage; Kamer and Som inserted a graft into the lower rim of the thyroid cartilage; and Opheim, Sawashima, and Tucker placed a graft between the thyroid cartilage and its inner perichondrium^41, 42^. By contrast, we inserted the graft obliquely through the thyroid cartilage via a created window. The graft was then placed vertical to the alar cartilage to lock it in and give it favorable stability.

In this study, for implantation in medialization laryngoplasty, we excised a single piece of lunar-shaped thyroid cartilage suitable for augmentation and laryngeal function. The implant may be modified in size or shape following excision depending on disease severity and laryngologist experience. For successful outcomes, it is recommended that the surgical procedure be performed by a well-trained laryngologist with basic training in head and neck surgery. The surgeon must be able to manage perioperative and postoperative complications, such as compromising airway and postoperative hematoma or infection. Emergent tracheostomy is less likely but still possible. This surgical procedure can improve MPT, F0, jitter, voice grade, roughness, and breathiness significantly, but not shimmer, NHR, asthenia, and strain. The possible reason is that the surgery aims to correct the closure of the glottis but does not change the muscular strength and vocal cord mucosa. As a result of efficient glottis closure, the mucosa wave and subglottic pressure are increased.

A recent study reported the long-term inflammatory response to liquid injectable silicone, cartilage, and a silicone sheet^46^. The surgical procedure reported in our study, in which an autologous thyroid cartilage implant was inserted for medialization with a single surgical wound, might prevent adverse effects such as infection, inflammation, and tissue rejection. Although cartilage implants have been used in plastic surgery for years, they have rarely been used in voice surgery. Fresh autologous cartilage has a high survival rate and is an ideal plastic material that can be used without the risk of foreign body reactions. In addition, compared with homologous grafts and alloplastic implants, autologous grafts are relatively highly biocompatible and have lower rejection rates^47, 48^. Thyroid cartilage grafts are freely available, do not require the creation of a second surgical wound, and are suitable for any patient who does not have severe laryngeal trauma with thyroid cartilage damage.

The procedure is performed under local anesthesia and intravenous sedation. Patients with inspiratory stridor or shortness of breath should undergo immediate cartilage removal, followed by consideration for a smaller graft. Postoperative pain is not severe because of the minimally invasive nature of the surgery, and patients can eat after a few hours. In contrast to feminization laryngoplasty, the appearance of the laryngeal prominence is well-preserved.

Similar to the outcome of Isshiki type I thyroplasty reported in previous studies^6, 22, 25, 32–34, 40^, inserting a thyroid cartilage graft implant for medialization improved MPT and voice parameters. Moreover, all patients exhibited an uneventful postoperative course without infection, tissue rejection, or graft dislocation. Although patients did not achieve improved voice quality immediately following the procedure, the voice quality of most patients improved within 1 week. This might be similar to silastic medializations, in which patients typically have a “tight” voice due to edema or paraglottic tissue cartilage detachment before reaching a more normal long-term voice quality. The significant improvement in MPT observed in this study indicates that the surgery corrected the glottal gap directly.

On the basis of our experience on implantation, male patients are more satisfied with the result than female patients. The large and firm thyroid cartilages of male patients are ideal material for augmentation. For patients with large posterior glottal gaps and malpositioned arytenoids, thyroid cartilage implantation alone may not be sufficient to improve the voice. Therefore, adduction arytenopexy or AA—which is helpful in patients with large posterior glottal gaps secondary to malpositioned arytenoids—may be simultaneously performed^41, 49, 50^. Additionally, cricothyroid subluxation can improve voice pitch, and this is controlled by the cricothyroid muscle^51^. After the aforementioned procedures, the glottal gap will have been significantly reduced. If a patient was still unsatisfied with their voice function, injection laryngoplasty is a safe and feasible therapy for improving their voice quality^52^. In this study, four unsatisfied female patients underwent hyaluronic acid intracordal injection after cartilage implantation. Three of them were finally satisfied with their voice, but one patient still suffered from poor swallowing function and hoarseness. This patient had previously undergone brain stem surgery because of a tumor. Neither thyroplasty nor an intracordal injection helped her significantly, but even the glottal insufficiency was completely improved after surgery.

This study has some limitations. First, because of the retrospective design of this study, analysis was limited to the data previously recorded in patients’ medical records. Second, the average follow-up duration was 21.4 months, resulting in a lack of data on the long-term voice outcome. Third, the sample size was small. However, this study proved that autologous thyroid cartilage graft implantation in medialization laryngoplasty achieved a favorable outcome and acceptable safety. To study the long-term efficacy of the surgery, future research should recruit more patients with longer follow-up.

In conclusion, autologous thyroid cartilage graft implantation in medialization laryngoplasty for treating UVFP can medialize the paralyzed vocal cord and minimize the glottal gap. Autologous thyroid cartilage implants are available for most patients and can improve their voice quality. In this study, significant improvements in MPT, F0, jitter, voice grading, roughness, and breathiness were observed in patients with UVFP who underwent this surgical procedure. This surgical procedure is a feasible approach for treating UVFP, is simple and safe, and produces acceptable results.

## Electronic supplementary material

Supplementary Information

Surgical procedure

Voice improvement

Raw data
