# Supplementary material for: Autologous thyroid cartilage graft implantation in medialization laryngoplasty: a modified approach for treating unilateral vocal fold paralysis
Source: Sci Rep. 2017 Jul 6;7:4790. doi: 10.1038/s41598-017-05024-6 (PMC5500551; doi:10.1038/s41598-017-05024-6)

# Title Page for Supplementary Information

# Autologous thyroid cartilage graft implantation in medialization laryngoplasty: a modified approach for treating unilateral vocal fold paralysis

Ming-Shao Tsai, M.D.^1,2^, Ming-Yu Yang, Ph.D.^3^, Geng-He Chang, M.D.^1^, Yao-Te Tsai, M.D. ^1^, Meng-Hung Lin, Ph.D.^2^, Cheng-Ming Hsu, M.D., F.A.C.S.^1,3,4,*^

^1^Department of Otolaryngology – Head and Neck Surgery, Chiayi Chang Gung Memorial Hospital, Chiayi, Taiwan

^2^Center of Excellence for Chang Gung Research Datalink, Chiayi Chang Gung Memorial Hospital, Chiayi, Taiwan

^3^Graduate Institute of Clinical Medical Sciences, College of Medicine, Chang Gung University, Taoyuan, Taiwan

^4^School of Traditional Chinese Medicine, College of Medicine, Chang Gung University, Taoyuan, TaiwanRunning title: Thyroid cartilage graft for treating unilateral vocal paralysis

*Correspondence author: Cheng-Ming Hsu

Department of Otolaryngology – Head and Neck Surgery, Chiayi Chang Gung Memorial Hospital, Chiayi, Taiwan

No 6, Sec. West, Jiapu Rd., Puzi-City, Chiayi County, Taiwan

E-mail: scm0031@cgmh.org.tw

Tel: +886-5-362-1000 ext. 2076 FAX: 886-5-362-3002

**The Supplementary Information of the study are listed in the following pages:**

| Title (Uploaded File Name) | Legend |
| --- | --- |
| Figure S1 | Twelve key steps of surgical procedure. |
| Video - surgical procedure | Demonstration of surgical procedure. |
| Video - voice improvement | Comparison of preoperative voice and postoperative voice. |


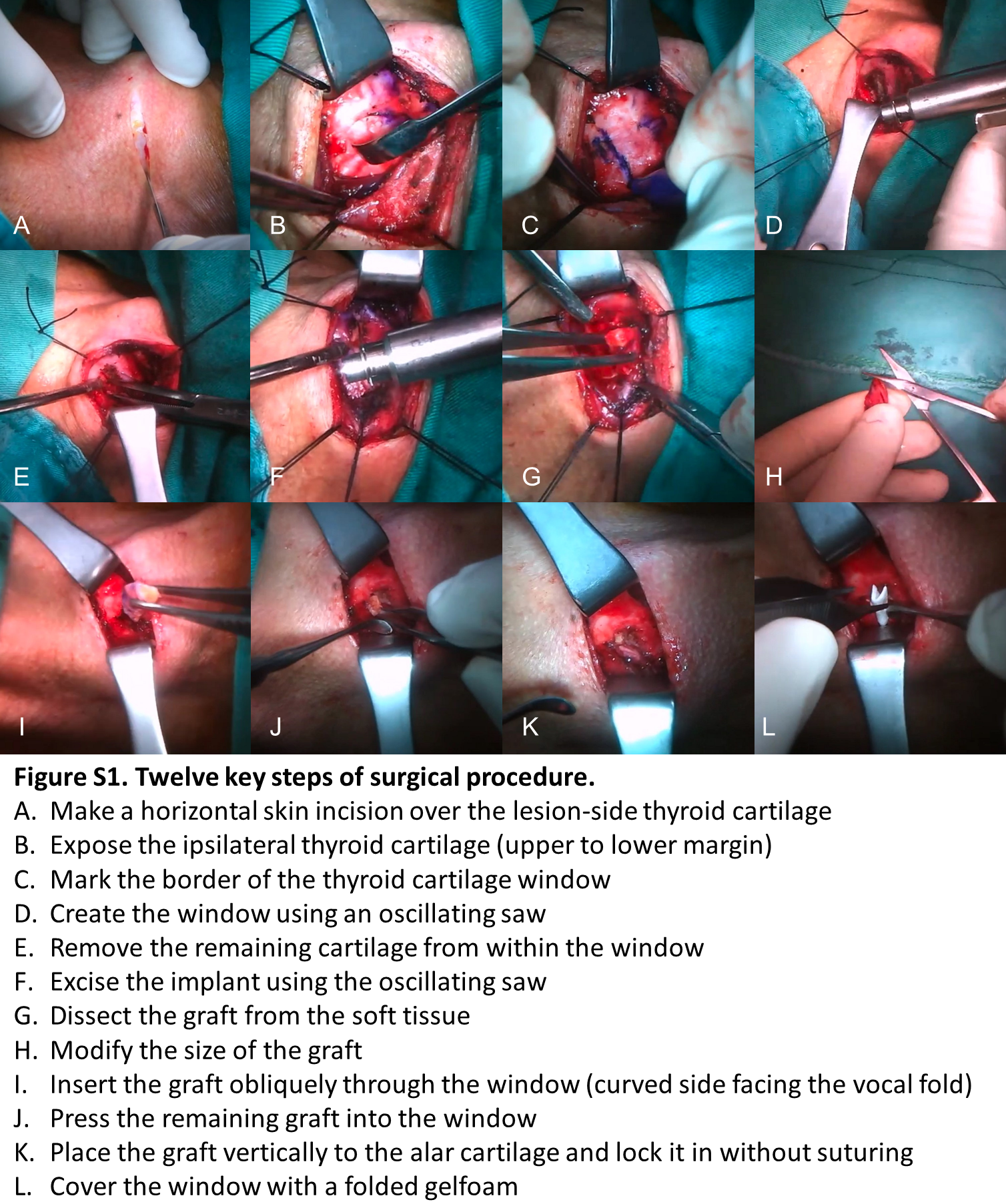

Supplement: Supplementary file 1 — Supplementary Information [file 41598_2017_5024_MOESM1_ESM.doc]
